# Predictors for vaccine hesitancy among nursing students in Namibia

**DOI:** 10.4102/jphia.v15i1.683

**Published:** 2024-11-05

**Authors:** Rebekka N. Gabriel, Alfeus D. Kashiva, Ottilie N. Shikesho

**Affiliations:** 1 Department of Community and Mental Health, Faculty of Health Sciences and Veterinary Medicine, University of Namibia, Windhoek, Namibia; 2 Department of Community Health, Faculty of Health Sciences, University of Namibia, Windhoek, Namibia; 3 Department of Public Health, Faculty of Health Sciences, University of Namibia, Windhoek, Namibia

**Keywords:** COVID-19, healthcare workers, hesitancy, nursing students, university students, vaccination

## Abstract

**Background:**

Healthcare workers (HCWs) are a priority group targeted for vaccination because they are greatly exposed to infectious agents.

**Aim:**

To determine the prevalence of coronavirus disease 2019 (COVID-19) vaccination and associated risk factors for vaccine hesitancy to recommend vaccine uptake strategies among student nurses.

**Setting:**

The study was carried out at the University of Namibia, main campus in Windhoek.

**Methods:**

A cross-sectional study was undertaken using a proportionate stratified sampling method. The total sample for the study was 509 students. Statistical Package for the Social Sciences (SPSS) 29 was used to analyse data. Frequencies and proportions were calculated. Bivariate analysis and multiple logistic regression were conducted to pronounce the predictors for vaccine hesitancy.

**Results:**

Only 31.40% students were vaccinated against COVID-19. Out of the vaccinated, 41.80% (*p <* 0.001) were previously infected with COVID-19. Participants who were not HCWs before nursing school were most likely not to be vaccinated (*p <* 0.001). Most unvaccinated students were hesitant because of a lack of information about the COVID-19 vaccine’s side effects (50.36%) and not knowing its effectiveness (38.13%). A total 60.80% of the participants believed a vaccine was needed to overcome the pandemic.

**Conclusion:**

We recommend the creation of targeted awareness campaigns that shed more light on the benefits of vaccination and vaccine side effects.

**Contribution:**

The study identifies key factors that can be targeted to change student nurses’ behaviour towards vaccination and offers valuable data that can inform preparedness and response strategies for future pandemics in Namibia and similar regions.

## Introduction

Vaccination is one of the most effective ways to control infectious diseases.^1^ While most of the population may easily accept and get vaccinated as per schedule, there remain individuals or mass groups who are against vaccination.^2^ Vaccine hesitancy is defined as the refusal of vaccination despite the availability of vaccines and accessibility of vaccination services.^3^ Vaccine hesitancy is a global problem that has many layers and faces.^3,4^ It can range from delayed acceptance of vaccination, complete refusal or taking the vaccine but with doubt concerning its safety and benefits.^5^ Over the past decades, there have been vaccine controversies, fuelled by adverse events (AEs) following immunisation and diminished confidence in vaccines and science.^5,6^ In addition, vaccine hesitancy can be aggravated by other factors particularly the novel nature of a disease, misinformation regarding the benefits of a vaccine, composition and anti-vaccination messages on mass media,^4,7,8^ among others. In terms of coronavirus disease 2019 (COVID-19), anti-vax groups were quick to surface with anti-vax messages, statements, myths, conspiracy theories, misconceptions and questions about how speedy the vaccine was developed, further sending huge masses into panic and confusion about getting vaccinated. The controversies, often powered by the media, have a negative impact on vaccination acceptance among the general population.^6^ This has not spared healthcare workers (HCWs), including student nurses.

Because of the nature of their work, HCWs are commonly named among the priority groups targeted for vaccination.^9^ Not only are they greatly exposed to infectious agents, but sick HCWs may also transmit diseases to their patients.^10^ However, it was noted that during pandemics, many HCWs have remained sceptical about getting vaccinated despite the magnitude of the pandemic, the COVID-19 pandemic being the classical example.^11^ Healthcare workers are a reliable source of information; hence, vaccine acceptance may mean the likelihood of recommending vaccination to the masses. It has been observed that hesitant HCWs were less likely to recommend vaccination to their clients and when they do, it is with reduced faith and confidence as compared to non-hesitant HCWs.^12^ Healthcare workers were also found to be less likely to recommend vaccination when AEs were likely to manifest, when they had less information on the vaccine or when they were not comfortable explaining the benefits and risks to clients.^5^

While the COVID-19 pandemic has been declared over globally, Namibia is currently experiencing a spike in cases in central-southern Namibia, with over 20 new cases reported in one district in a week^13^ where University of Namibia (UNAM) nursing students often conduct training. Despite this, little is known with regard to vaccine hesitancy in Namibia, especially among HCWs, medicine and nursing students. Moreover, the drivers for vaccine hesitancy have not been well explored in the country particularly among nursing students. This study aimed to determine the prevalence of COVID-19 vaccination among nursing students at the UNAM and to assess the factors associated with vaccine hesitancy with an overall aim to recommend vaccine uptake strategies among student nurses in Namibia.

## Research methods and design

A cross-sectional study was undertaken between October 2022 and November 2022 at the UNAM main campus. A proportionate stratified random sampling strategy was adopted, whereby classes were regarded as strata. The university has seven nursing classes composed of diploma and degree students. A sample was collected from each class using a random sampling method. The sample size was determined using the Slovin’s formula:

$$\text{Sample size }(n)=N/(1+N{\text{e}}^{2}),$$ [Eqn 1]

in which *n* = sample size, *N* = the estimated population size, with a margin error of 5%. The total population was 633 students. A total of 509 students were drawn as a sample, 91 from the 1st year degree class, 81 from the 2nd year degree class, 77 from the 3rd year degree class, 76 from the 4th year degree class, 78 from the 1st year diploma class, 46 from the 2nd year diploma class and 60 from the 3rd year diploma class. The proportionate stratified sampling technique was adopted to ensure the representativeness of the sample per class. A self-administered questionnaire was used to collect data. The questionnaire sought demographic characteristics of participants (age, sex, marital status and religion), information on COVID-19 vaccination status and predictors for vaccine hesitancy. It took roughly 15 min – 20 min to complete the questionnaire. Content validity was ensured via expert review.

We used Statistical Package for the Social Sciences (SPSS) version 29 (IBM, Armonk, New York, United States [US]) to analyse the collected data. At first, frequencies and proportions were calculated to pronounce the demographic characteristics of study participants. We then calculated the prevalence of COVID-19 vaccination and predictors for vaccine hesitancy. We conducted a bivariate analysis and multiple logistic regression to pronounce the predictors for vaccine hesitancy. A *p*-value of less than 0.05 was considered statistically significant.

### Ethical considerations

Ethical approval to conduct this study was obtained from the University of Namibia School of Nursing Ethical Committee (No. SoNEC 56/2022). Written consent was obtained from participants prior to data collection. To maintain confidentiality, no identifying information was collected from the participants.

## Results

A total of 467 responses were recorded. This means the study had a non-response rate of 8.25% (*n* = 42). In all, 18 responses (4%) were excluded because of gross incompleteness of the questionnaires.

### Demographic characteristics and COVID-19 vaccination prevalence

**FIGURE 1:**
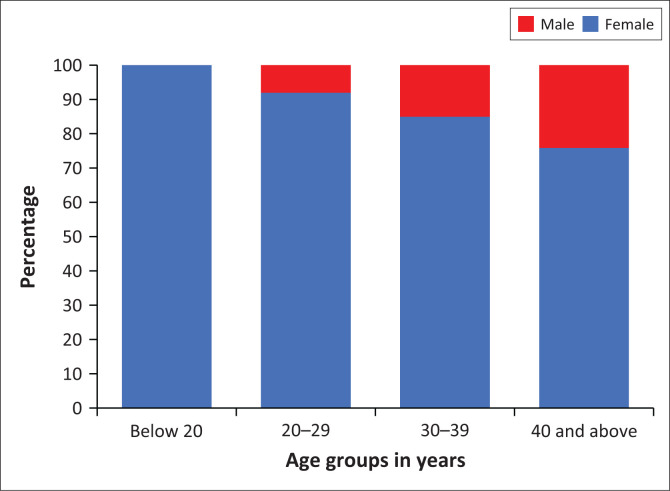
Sex distribution per age group of participants.

Of all the participants, 10.47% (*n* = 47) were males, while 89.53% (*n* = 402) were females. The median age was 26.73 years, while the mode was 25.81 years (see Figure 1).

Out of the 449 participants, only 31.40% (*n* = 141) of students were vaccinated against COVID-19. Of the vaccinated students, 41.80% (*p* < 0.001) were previously infected by COVID-19. To further assess vaccine acceptability, unvaccinated participants (*n* = 308) were asked whether they were willing to get vaccinated. Only 30 (6.7%) participants indicated their willingness to get vaccinated (*p* < 0.001). Participants who have never been HCWs were most likely not to be vaccinated as compared to those who have prior experience. Table 1 probes more into the demographics and vaccine prevalence.

**TABLE 1:** Demographic characteristics and COVID-19 vaccination prevalence.

Characteristic	Vaccinated	Not vaccinated	COR	95% CI	AOR	95% CI
**Gender**						
Male	22	25	Ref	-	Ref	-
Female	119	283	2.09	1.13–3.85	1.5	0.77–2.94
**Age group**						
Below 20	7	32	Ref	-	Ref	-
20–29	47	201	7.48	2.46–22.69**	5.07	1.21–21.18
30–39	69	64	6.99	3.09–15.80**	3.83	1.25–11.70
40 and above	18	11	1.51	0.66–3.45	1.47	0.60–3.60
**Marital status**						
Single	102	270	Ref	-	Ref	-
Married	34	31	0.34	0.20–0.59**	1.12	0.58–2.15
Divorced	0	2	> 100	0.00–0.00	> 100	0.00–0.00
Widowed	2	0	0.00	0.00–0.00	0.00	0.00–0.00
Cohabitating	3	5	0.63	0.148–2.68	1.36	0.26–6.97
**Class**						
Degree 1	17	64	Ref	-	Ref	-
2	13	61	1.24	0.55–2.78	1.41	0.61–3.25
3	16	62	1.02	0.47–2.21	1.28	0.57–2.87
4	15	44	0.77	0.35–1.72	1.17	0.50–2.75
Diploma 1	43	19	0.11	0.05–0.25**	1.38	0.24–7.96
2	19	19	0.26	0.11–0.61*	3.41	0.56–20.73
3	18	39	0.57	0.26–1.24	10.4	1.72–62.79*
**HCW before?**						
Yes	86	77	Ref	-	Ref	-
No	55	231	4.69	3.06–7.17**	7.87	3.04–20.34**

COR, crude odds ratio; AOR, adjusted odds ratio; Ref, reference; CI, confidence interval; HCW, healthcare worker.

* , *p* < 0.05;

** , *p* < 0.001.

### Factors for general and COVID-19 vaccine hesitancy

Coronavirus disease 2019 vaccine was determined by posing the question, ‘*Why do you not want to be vaccinated against COVID-19?*’ to participants who were not vaccinated and who replied ‘No’ or ‘Not sure’ when questioned whether they were willing to get vaccinated. Participants were required to pick one or more reasons that apply to them. Whereas, all participants, regardless of their COVID-19 vaccination status were asked to select the factors that would be important for them to be confident in taking and/or recommending a vaccine. This was done to determine factors that may affect general vaccine hesitancy. They could pick one or more factors.

The lack of information about the COVID-19 vaccine’s side effects was the top factor causing hesitancy (50.36%). Participants also indicated that not knowing the importance and effectiveness of the COVID-19 vaccine led to them being hesitant (Table 2). Majority (91.09%) of the participants indicated hesitancy towards new vaccines. However, only two other factors were given for hesitancy, one being patients still getting sick or dying of COVID-19 after vaccination.

**TABLE 2:** Factors for vaccine hesitancy.

Variable	*n*	%
**Predictors for COVID-19 vaccine hesitancy (*n* = 278)**		
Once vaccinated, I will still have to live my life with COVID-19 restrictions	47	16.90
I am not fully informed about the COVID-19 vaccine’s side effects	140	50.36
I do not know the importance and effectiveness of COVID-19 vaccine	106	38.13
Too many undispersed conspiracy theories	45	16.19
I am not scared of the COVID-19 disease	25	8.99
**Predictors for general vaccine hesitancy (*n* = 449)**		
The vaccine should not be new	409	91.09
I should be able to confidently relay information and/or facts to the public	253	56.34
I should have confidence in the vaccine’s safety and efficacy	377	83.96
I should be fully informed of the vaccine’s side effects	389	86.63
Conspiracy theories should be discredited	185	41.20
Others (Specify)	2	0.44

COVID-19, coronavirus disease 2019.

### Beliefs, opinions, and attitudes of participants regarding COVID-19 vaccination

In this section, ‘most definitely’ and ‘definitely’ were regarded as agreeing with the statements, while ‘definitely not’ and ‘most definitely not’ in disagreement. The majority of participants (84.84%) believed that COVID-19 is real. The results also revealed a strong fear of the COVID-19 vaccine side effects (61.47%). Despite this, majority of participants (60.80%) did agree that the COVID-19 vaccine was needed to overcome the pandemic. In the same vein, many participants (82.40%) revealed that they trust the HCWs (Table 3).

**TABLE 3:** Beliefs, opinions and attitudes regarding vaccination.

Statement	Most definitely	%	Definitely	%	Definitely not	%	Most definitely not	%
COVID-19 disease is not real	29	6.45	39	8.68	132	29.39	249	55.45
I am scared of COVID-19 vaccine side effects	142	31.63	134	29.84	94	20.94	79	17.59
COVID-19 vaccine is needed to overcome the pandemic	111	24.72	162	36.08	129	28.73	47	10.47
COVID-19 was created by the whites to control black community	31	6.90	53	11.80	158	35.19	207	46.10
COVID-19 vaccine is connected to 5G network	20	4.45	37	8.24	189	42.10	203	45.21
My religion prohibits me from vaccines	12	2.67	14	3.12	135	30.07	288	64.14
I do not trust the HCWs	30	6.68	49	10.91	152	33.85	218	48.55
COVID-19 is politically and economically influenced	104	23.16	123	27.39	101	22.49	121	26.95

COVID-19, coronavirus disease 2019; HCW, healthcare worker.

## Discussion

This study aimed to determine the prevalence for COVID-19 and assess factors associated with hesitancy among student nurses. Only 31.40% (*n* = 141) of the participants were vaccinated against COVID-19. In addition, only 6.7% of the non-vaccinated expressed willingness to be vaccinated. Various studies have reported low prevalence among HCWs and university students in numerous countries.^14,15,16^ In contrast, some studies found that more than half (> 50%) of the participants were vaccinated or intended to be vaccinated for numerous reasons such as having chronic illnesses, protection from the virus, believing that the vaccine can prevent further spread in the community and that the getting vaccinated can end the pandemic.^17,18,19,20^ This study found no significant difference in age, gender and marital status. This was in agreement with a systematic review that assessed the global prevalence of COVID-19 vaccine prevalence among medical students.^18^

Although age yielded no significant difference, the study found that 3rd year diploma students were more likely to be vaccinated when compared to their counterparts. The diploma course at UNAM is a bridging course for enrolled nurses (EN) to upgrade to convert to registered nurses (RN). This means this class consists of older students who were HCWs before returning to school. The results are in agreement with studies done in Nigeria, which reported that older people are more likely to accept COVID-19 vaccines than younger people.^21,22^ This may be because, third-year diploma students have more years of education, exposure to the medical field and knowledge that may affect the uptake of vaccines. The study results revealed that those who had previously suffered from COVID-19 were likely to be vaccinated. This is consistent with a study done in Sudan, where previously infected medical students were more likely to accept vaccination.^19^ In addition, a systematic review by Limbu and Huhmann also indicated previous infection as a strong indicator for vaccination.^23^ Contradicting results were reported in Texas, where students from various health disciplines previously infected by COVID-19 were more hesitant towards vaccination than those who were never infected.^24^ The findings in Texas might have been attributed to studies that suggest the unlikeliness of previously infected people to benefit from COVID-19 vaccination.^25,26,27^

Upon assessing the factors for COVID-19 vaccine hesitancy among those hesitant to be vaccinated, more than half the participants indicated not being informed about the vaccines’ side effects. These results bring forth two concerns: the lack of information on side effects and the fear of side effects. The lack of information has been reported as a factor by studies conducted in Egypt and Cameroon.^28,29^ Healthcare workers are among the groups targeted for early vaccination. This means that in the outbreak hit with an overall lack of information and confusion, HCWs had it worse because of early vaccination requirement regardless of very little information available at the time. Participants in this study were also afraid of the side effects. These results were similar to other studies whose participants worried that side effects may be more detrimental to their health compared to acquiring the virus.^17,30,31^ Moreover, students reported not being informed about the vaccines’ effectiveness. This is in line with a study done among university students across Pakistan.^32^ Unsurprisingly, these parameters may have only been pointed out as predictors because of the novelty of the virus. Because the virus is new, there was a lack of sufficient and a lack of access to information during the period of vaccination roll-out.

In this study, the novelty of vaccines was also a factor for hesitancy, as when questioned ‘What else would be important for you to be confident in taking/recommending a vaccine?’, 91.09% of the total participants pronounced that the vaccine should not be a new one. Similarly, to COVID-19 vaccine hesitancy, participants wished to be fully informed about the side effects and have no lack of confidence in the safety and efficacy of the vaccine. These results can be a good indicator of how much still needs to be done in Namibia in educating the HCWs and healthcare students on the vaccines, especially with the re-emergence of COVID-19 cases. The lack of confidence in the vaccine as well as fear of the side effects have been stated as major factors for hesitancy globally. One can consider education and reassurance as crucial factors to improving vaccine hesitancy, not just for COVID-19 but for future vaccine-preventable outbreak responses too. This is not a surprising outcome especially given the novelty nature of the vaccines and that they were manufactured in a short timeframe. With the emergence of COVID-19 came lots of conspiracy theories on the disease and vaccine that were further spread by social media. Almost half (41.8%) of the participants wished for these theories to be dispersed. A study conducted in Saudi Arabia reported a lack of knowledge among medical students, with 97.9% believing that the COVID-19 vaccine involved conspiracy. Taking these statistics into consideration, medical experts may rethink the use of social and mass media, and the influence they have on the general population. Similarly, the promotion of facts in the media is vital to ensure effective education of the masses.

Upon assessing their beliefs, opinions and attitudes, the study found that majority (84.84%) of the participants believed that COVID-19 is real, and more than half (60.80%) agreed that a vaccine was needed to overcome the outbreak. Despite the positive responses, the overall vaccination prevalence among this group was low. This is an indicator that positive beliefs and opinions do not necessarily equate to positive attitudes, especially amid doubts and a lack of confidence.

## Conclusion

In conclusion, COVID-19 prevalence among nursing students at UNAM is low. Student nurses who were HCWs prior to nursing school were more likely to be vaccinated. Worry about side effects, a lack of confidence in the vaccine’s safety and efficacy, and a vaccine being novel were termed the major factors for vaccine hesitancy. Despite the vaccine prevalence being low, participants agreed that COVID-19 is real and a vaccine is needed to halt the outbreak.

As Namibia reports a spike in new COVID-19 cases, the results of this study may be useful in designing HCWs-targeted awareness campaigns that shed more light on the benefits of vaccination and vaccine side effects. Special emphasis should be paid to spreading information on how vaccine testing was done to validate the safety and efficacy of the vaccines given that production happened in a limited time frame. This may boost vaccine reception not only among HCWs, but also the general population.
